# Effect of nanosilica on the hydrological properties of loess and the microscopic mechanism

**DOI:** 10.1038/s41598-024-64280-5

**Published:** 2024-06-13

**Authors:** Li Wang, Qian Liu, Xi-An Li, Biao Qin, Bo Hong, Jianfeng Shi

**Affiliations:** 1 College of Architecture and Civil Engineering, Xi’an University of Science and Technology, Xi’an, 710054 Shaanxi China; 2 First Hospital of Northwestern University, Xi’an, 710043 Shaanxi China; 3 College of Geological Engineering and Geomatics, Chang’an University, Xi’an, 710054 Shaanxi China; 4 Key Laboratory for Geohazards in Loess Area, MNR, Xi’an Center of China Geological Survey (Northwest China Center for Geoscience Innovation), Xi’an , 710119 Shaanxi China; 5 China Jikan Research Institute of Engineering Investigations and Design Co. Ltd, Xi’an, 710016 Shaanxi China

**Keywords:** Loess, Nanosilica, Collapsibility coefficient, Soil water characteristic curve, Micromechanism, Environmental sciences, Natural hazards

## Abstract

Loess areas, such as the Loess Plateau, are characterized by a fragile ecological environment, high soil erosion, and frequent geological disasters due to the unique hydrological properties of loess (e.g., collapsibility and permeability). Therefore, the loess must be stabilized for use in engineering construction. Traditional stabilizers (lime, cement, and fly ash) cause environmental problems, such as soil salinization and greenhouse gas emissions. Therefore, this study investigated the effect of nanosilica on the hydrological properties of loess and the microscopic mechanism. Different nanosilica contents (0.2%, 0.4%, 0.8%, 1%, and 3%) were added to loess sample, and the particle size distribution, Atterberg limits, collapsibility, and soil water characteristics were analyzed. The results revealed the following. The addition of nanosilica changed the particle size distribution, liquid limit, plastic limit, and plasticity index of loess. After the addition of nanosilica with different contents, the loess collapsibility coefficient curve shifted downward, the soil water retention curve shifted upward, and the unsaturated permeability coefficient curve shifted downward. The pores between particles were filled, and the number of large and medium pores and the pore connectivity were lower after the nanosilica addition. The surface of the coarse particles adsorbed more fine particles, and a large number of micro-aggregates or clay aggregates were present in the pores between particles. In conclusion, the environmentally friendly material nanosilica can be used to improve the hydrological properties of loess, which is applicable to alleviating soil erosion and preventing geological disasters on the Loess Plateau.

## Introduction

Loess is widely distributed in the arid and semi-arid regions of Asia, Europe, North America, and South America. Loess areas cover 13 million km^2^ worldwide, accounting for 9.3% of the total land area^1–5^. The Loess Plateau of China is the largest loess region with the most complete stratigraphic development and largest sedimentary thickness in the world. It is located in northwest China and extends 1000 km from east to west and 800 km from north to south. It includes most areas east of Qinghai Province, north of the Qinling Mountains, west of the Taihang Mountains, and south of the Great Wall^1,4,6–9^. Loess is a porous and weakly cemented quaternary deposit formed predominantly by accumulated wind-blown dust, resulting in unique engineering and geologic properties^5,7,10^. Macroscopically, the advantages of loess are numerous seepage channels, such as joints, fissures, and sinkholes, which affect loess’ structural properties. Microscopically, the loose and porous structure is prone to deformation when encountering water, resulting in unfavorable properties^11–14^. Loess has high strength and stability at a low water content. As the water content increases, it exhibits unique hydrological properties, such as collapsibility and disintegration, significantly weakening structural connectivity and strength^7,10^. The hydrological properties of loess affect its water storage and transportation capacity, i.e., water retention and permeability, and its deformation and strength, such as plasticity, collapsibility, disintegration, and resistance^15^. The collapsibility and permeability are the primary characteristics affecting loess’ use in construction projects. The ecological and geological environment of the Loess Plateau is highly fragile due to loess’ unique hydrological properties. Thus, this region has very high soil erosion levels and is prone to geological disasters^16–20^. Therefore, it is necessary to develop improvements to enhance the hydrological properties of loess to mitigate soil erosion and prevent or control geological disasters on the Loess Plateau.

Many Chinese and international scholars have researched strengthening methods for loess, including mechanical curing methods (vibration, rolling, and dynamic compaction), traditional chemical curing methods (injection or chemical additives, such as lime, cement, and fly ash), and non-traditional curing methods^21–23^. Mechanical reinforcement is the preferred method in engineering practice but has the disadvantage of generating significant vibration and noise during compaction, potentially degrading adjacent slopes and buildings^24^. Therefore, an increased number of researchers and engineers have focused on developing alternative reinforcement methods.

Traditional chemical curing utilizes lime, cement, fly ash, and their mixtures with pozzolanic materials. Four main reactions/processes (exothermic hydration, ion exchange, structural change, and the generation of new compounds) occur when these products are combined with loess^25–29^. The curing of loess by adding lime, cement, and fly ash includes two stages: physical and chemical changes (water content and density) and changes in mineral composition and microstructure. This process improves the physical and mechanical properties of loess^26,27,30^. However, the solidification of loess by chemical curing increases the soil pH and salinity, resulting in a more alkaline and saline environment of the solidified loess, causing significant environmental problems^29,31,32^. Therefore, researchers and engineers have investigated non-traditional curing materials that are more environmentally friendly and effective to replace traditional chemical curing materials.

Nanomaterials have recently been widely used in geotechnical engineering and geohazards to improve soil stability due to their unique particle structure, large specific surface area, and high surface activity^33,34^. Pham and Nguyen^35^ found that polyethylene glycol (PEG)-coated silica nanoparticles inhibited clay swelling. Bahmani et al.^36^ investigated the influence of nanosilica on residual soil stability and found that the addition of nanoparticles had positive influences on the compaction, hydraulic conductivity, and compressive strength of residual soil. Ng and Coo^37^ investigated the hydraulic conductivity of nanomaterials mixed with clay. They observed that the nanomaterials clogged the clay’s pores, decreasing its hydraulic conductivity. Ren and Hu^38^ analyzed the effect of nanosilica on the physical and mechanical properties of silt and found that nanosilica increased the liquid limit, plastic limit of clay and its uniaxial compressive strength. Iranpour^39^ studied the impacts of nanomaterials on soil collapse. The results showed that different nanomaterials had different effects on the responses of collapsible soils. Taha et al.^40^ found that nano-clay, nano-aluminum, and nano-copper additives limited the expansion and shrinkage of clay without changing the soil’s mineralogical properties. However, research on using nanomaterials to cure loess is very limited. Only a few scholars have studied the effect of nanoparticles on compaction, unconfined compressive strength, shear strength, microstructure, and mineral composition of loess^41–45^. Therefore, this study examines the addition of different nanosilica contents (0.2%, 0.4%, 0.8%, 1%, and 3%) to loess sample. Tests are conducted to evaluate the particle size distribution, Atterberg limits, collapsibility, and soil water characteristics and investigate the effect of nanosilica on the hydrological properties of loess and the micro-mechanism. The research results are applicable to the mitigation of soil erosion and the prevention and control of geological disasters on the Loess Plateau.

## Material and methods

### Test materials

**Figure 1:**
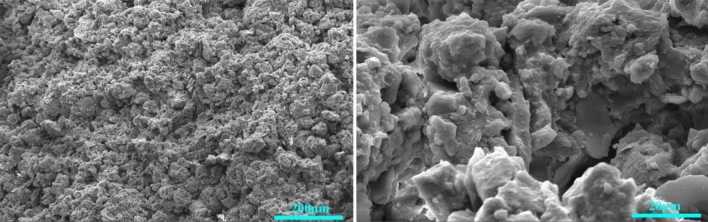
Scanning electron microscope images of loess samples at different magnifications.

**Figure 2:**
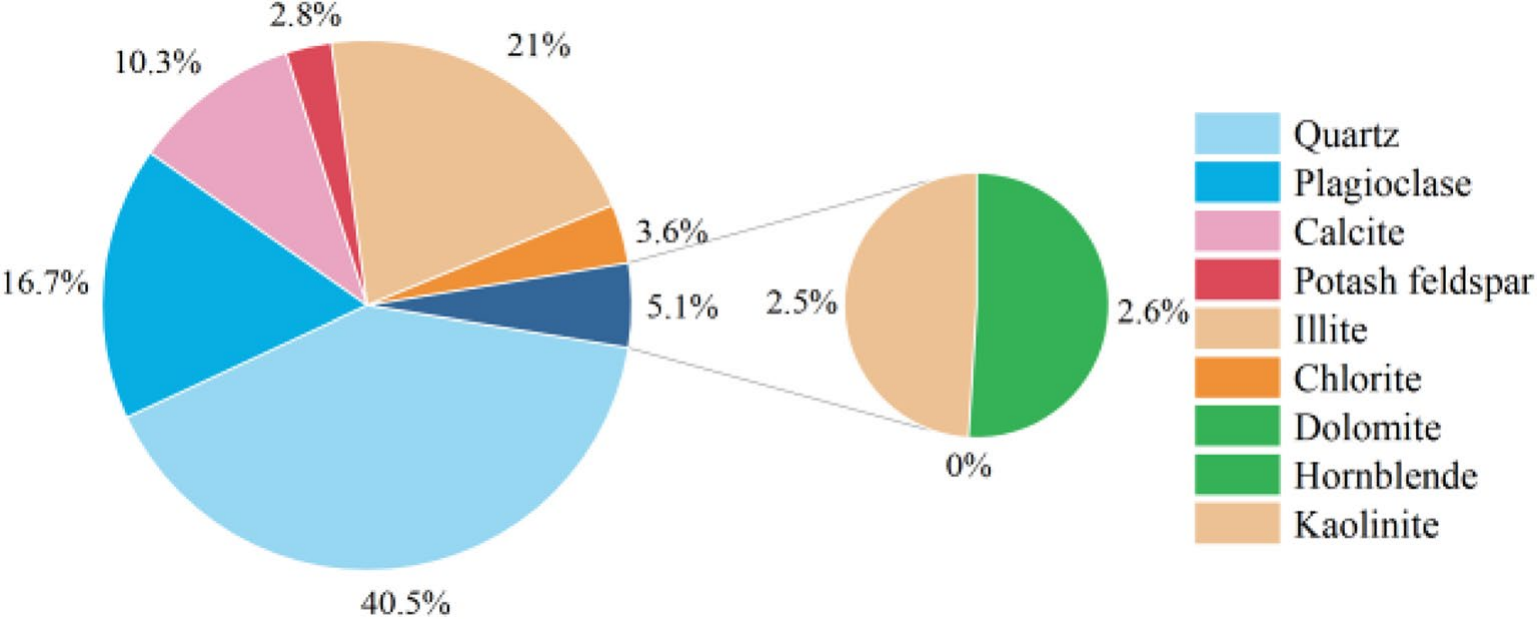
Mineral composition of loess samples.

**Figure 3:**
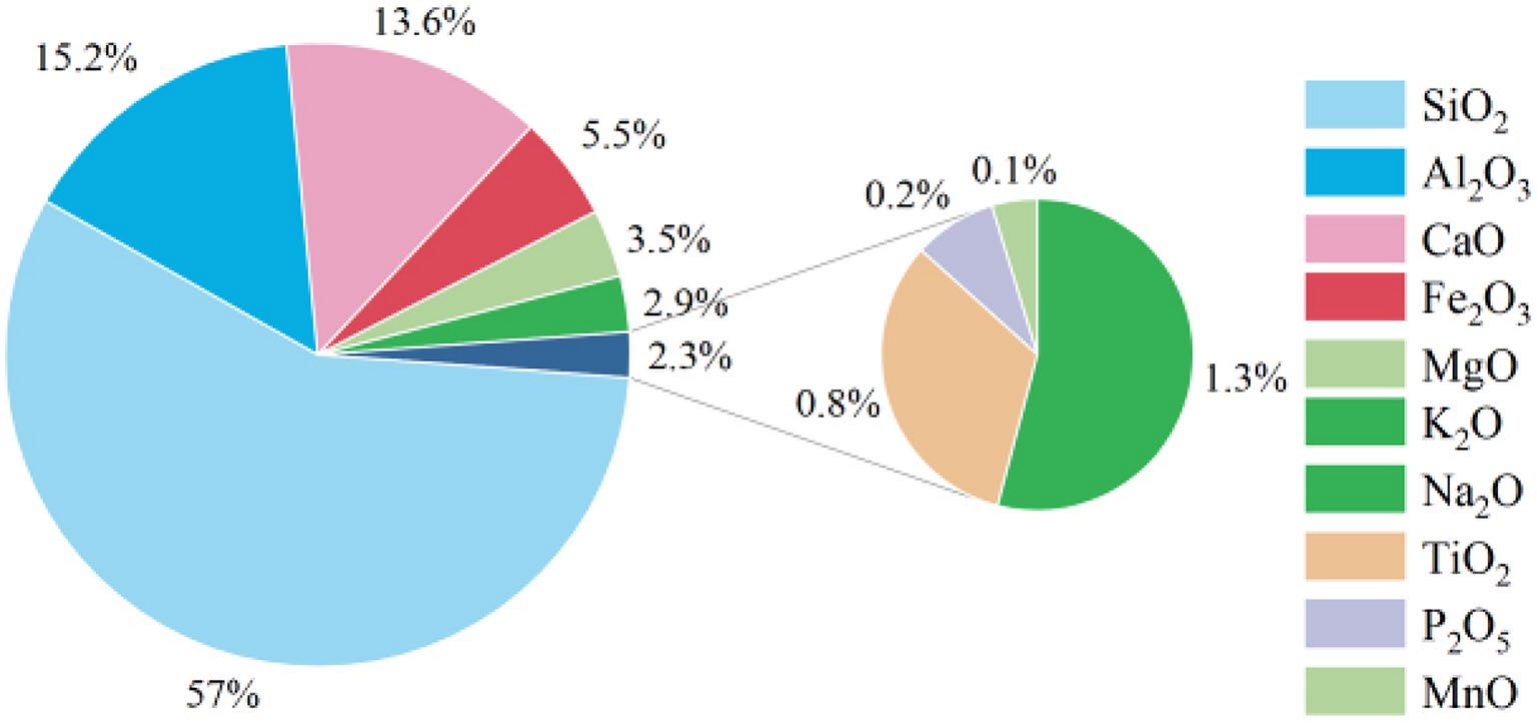
Chemical composition of loess samples.

The loess used in the test was collected from a natural steep slope near the Chan River in Xi’an City, Shaanxi Province, at a depth of about 4 m. It was Late Pleistocene loess (Q_3_ Malan loess). Manual excavation was used to prevent the disturbance of the samples. The extracted soil was cut into cylinders (diameter Φ ≈ 10 cm, height h ≈ 20 cm) on site, and the deposition direction of the soil samples was marked. The samples were sealed with black film and tape to prevent water loss and were labeled. Table 1 lists the basic physical properties of the loess specimen measured according to the Standard for the Geotechnical Testing Method of the P. R. of China (GB/T, 50123-2019, 2019)^46^. A Quanta FEG 450 scanning electron microscope was used to observe the microstructure. The results are shown in Fig. 1. X-ray fluorescence spectroscopy was used to test the chemical composition of the loess samples; the results are shown in Fig. 2. A Bruker ASX D8 X-ray diffractometer was used for the mineral analysis of the air-dried, powdered loess samples. The results are shown in Fig. 3.

**Table 1:** Basic physical properties of the loess specimens.

Specific gravity *Gs*	Water content *w*/%	Dry density *ρ*_*d*_/g cm^−3^	Plastic limit *w*_*P*_/%	Liquid limit *w*_*L*_/%	plasticity index *I*_*P*_	Particle composition		
						2–0.05 /mm	0.05–0.002 /mm	< 0.002 /mm
2.70	11.92	1.47	17.7	31.4	13.7	5.26	77.08	17.30

### Nanosilica

**Figure 4:**
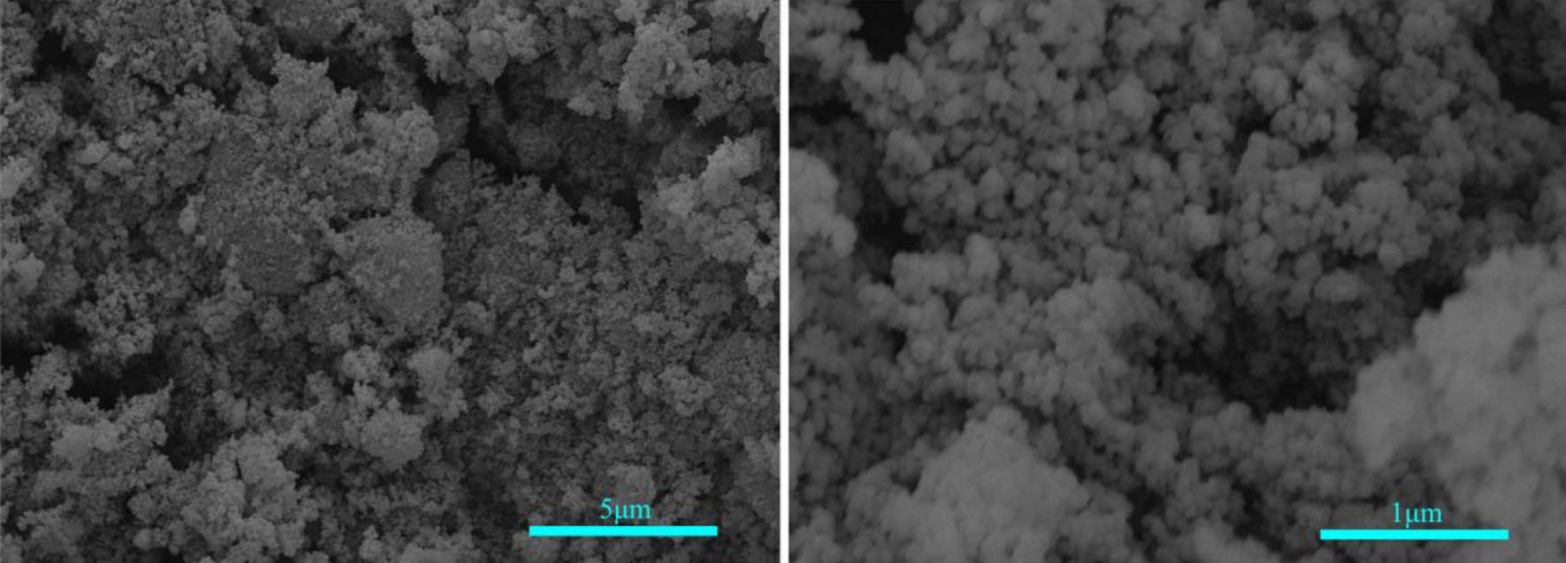
Microstructure morphology of nanosilica at different magnifications.

Due to their very high specific surface area (SSA) and charged active surface, nanoparticles can interact with other soil components, including liquid phases, cations, organic matter, and clay minerals, even when present in small amounts. Therefore, nanosilica significantly affects the soil’s microstructure and its physical, chemical, and engineering properties^33,34,47^. TSP-H10 nanosilica, manufactured by Nanjing Tianxing New Materials Co. Ltd, China, was selected for the test. Table 2 lists the physical and chemical properties of the material. Figure 4 shows the microscopic morphology of the nanosilica.

**Table 2:** Physical and chemical properties of the nanosilica.

Average particle size nm	Specific surface area m^2^/g	Bulk density g cm^−3^	Purity %	Color	pH
20	200	0.10	> 99	White	4–7

### Sample preparation

The sample preparation consisted of the following steps: (1) The undisturbed loess was air-dried and crushed, and impurities were removed by passing the loess sample through a sieve with a diameter of 2 mm. The sieved loess was dried in an oven at 105 °C. (2) The nanosilica and loess were thoroughly mixed by multiple sieving and mixing to obtain a homogeneous mixture containing 0.2%, 0.4%, 0.8%, 1%, and 3% (dry weight, by mass) of nanosilica and loess. The concentration setting of nano silica Refs.^23,34,39,42,43^. (3) The dry mixture was uniformly sprayed with an appropriate amount of distilled water to obtain the target moisture content. (4) The wet mixture was placed into a sealed plastic bag in a petri dish for 48 h to ensure an even water distribution. (5) The specimens were remolded. They had the same moisture content and dry density as the loess sample.

### Testing procedures

#### Geotechnical tests

In order to evaluate the effects of nanosilica on the hydrological properties of loess, several geotechnical tests including particle size distribution^46^, Atterberg limits^46^, collapsibility test^46^ were conducted on the loess soil samples.

#### Soil water characteristic curve

Common method for determining the soil water characteristic curve include the tensiometer, pressure film, and centrifuge methods. The latter is the most widely used due to its simplicity, time efficiency, and wide range of measurable suction^48,49^. The centrifuge was a CR21G high-speed refrigerated centrifuge (Hitachi Co., Japan) with a maximum speed of 11,400 rev/min. A ring knife was used to create a cylindrical sample (6 cm × 30 cm^2^), and the ring knife of the centrifuge reduced the size of the sample to 5 cm × 20 cm^2^. The sample was then sealed with plastic wrap. After soaking the samples in distilled water for 48 h, they were placed in the centrifuge to determine the soil water characteristic curve. The experimental procedures and calculations are described in Refs.^50,51^.

#### Unsaturated permeability coefficient

It is difficult to measure the permeability coefficient of unsaturated soils directly due to matric suction. Many scholars have proposed methods to calculate it indirectly. The van Genuchten model^52^ is suitable for various soil types and has been widely used in studies of loess^50,51^. Therefore, this model was used to fit the soil water characteristic curve. The unsaturated permeability coefficient was based on the saturated permeability coefficient and the soil water characteristic curves of loess with different nanosilica contents obtained from the centrifuge test. The details of the calculations have been described in Refs.^51,52^.

#### Microstructure observations

The sample preparation process for the analysis of the loess microstructure has been described in Ref.^5^. The specific process: (1) Take out the sample to be observed, used a blade or saw to cut a cylinder with a diameter of 1 cm and a height of 1.5 cm, and carving a ring of grooves in the middle, and then breaking the sample to obtain fresh section; (2) The cylinder sample was dried naturally at room temperature. (3) The sample was fixed on the target platform, and sprayed gold film using an ion sputtering instrument to ensure that the sample section had good electrical conductivity; (4) The sample was placed on the scanning electron microscope stage for microscopic observation and image capture.

## Results and discussion

### Particle size distribution

**Figure 5:**
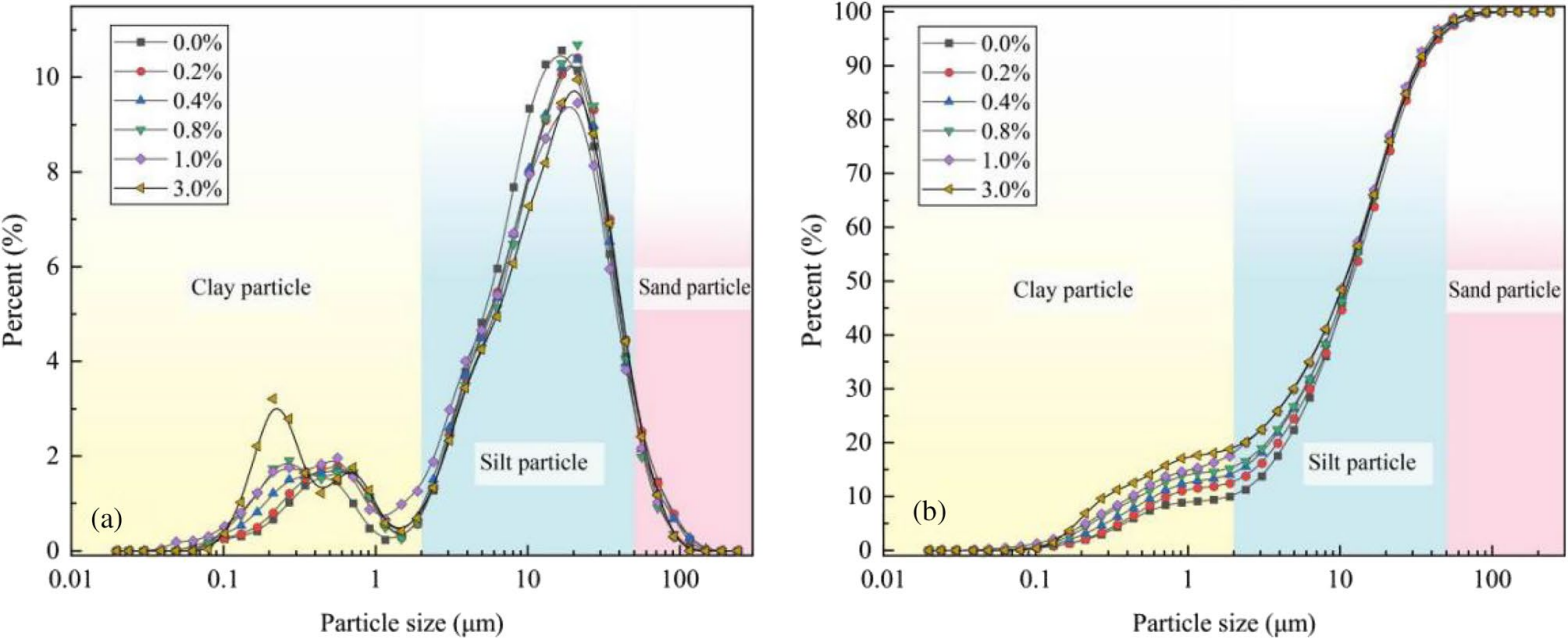
Effect of nanosilica addition on loess particle size distribution. (**a**) Frequency particle size distribution; (**b**) Cumulative particle size distribution. Classification of particle fractions based on Ref.^46^.

Figure 5 shows the particle size distribution curves of the loess samples with different nanosilica contents. Figure 5a presents the frequency particle size distribution. Most of the particles have sizes of 0.1–2 μm (clay) and 2–50 μm (silt), and the trends are similar for the different nanosilica contents. The content of fine particles increases, and the content of coarse particles decreases slightly with the increasing nanosilica content in the range of 2–50 μm. Figure 5b presents the cumulative particle size distribution for different nanosilica contents. The nanosilica addition primarily affects the distribution of loess particles in the range of 0.1–10 μm. The particle content in this range increases with the nanosilica amount. The content of particles smaller than 2 μm is significantly higher in the samples containing different amounts of nanosilica than in the untreated loess. The average particle size of the samples containing nanosilica particles is 20 nm (0.02 μm). The reasons are as follows. First, the nanosilica particles are not single dispersed particles but are aggregates (Fig. 4). Second, the nanosilica particles interact with micro—and nano-sized colloidal and clay particles in the loess, resulting in a change in the particle size distribution of loess (Fig. 5b), predominantly in that of the clay particles (Fig. 5a).

### Atterberg limits

**Figure 6:**
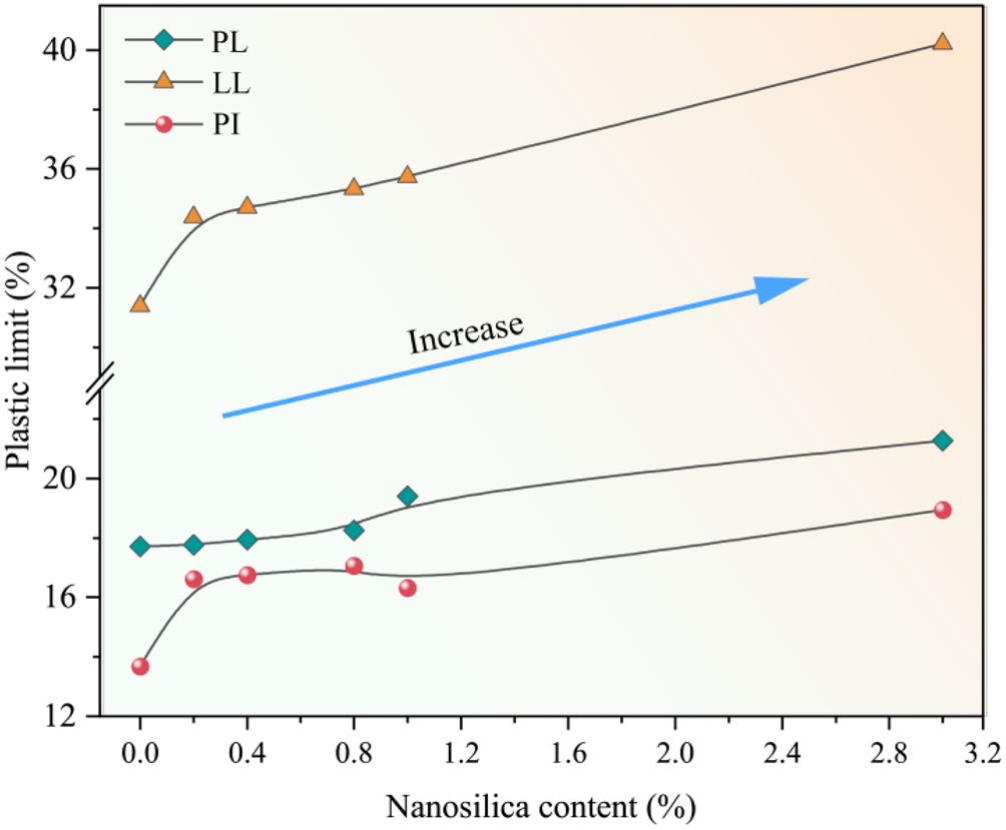
Effect of nanosilica addition on the Atterberg limits.

Figure 6 shows the liquid limit (LL), plastic limit (PL) and plasticity index (PI) of the loess samples with different nanosilica contents. The LL, PL, and PI increase with the nanosilica content. As the nanosilica content increases from 0 to 3%, the PL increases by 20%, the LL increases by 28%, and the PI increases by 38.8%. The main reasons are the following. (1) Nanosilica is a hydrophilic nanomaterial that attracts water molecules. Thus, the addition of nanomaterials increases the soil’s water-holding capacity. (2) Nanosilica has a larger SSA and higher cation exchange capacity (CEC) than loess sample, improving the water retention characteristics of loess and the chemical interaction between particles and water^33,34^. Our results are consistent with those obtained by other scholars^40,41^. However, in some studies, the PI was lower after the addition of nanosilica, which is the opposite of what we observed^47,53,54^.

### Collapsibility test

**Figure 7:**
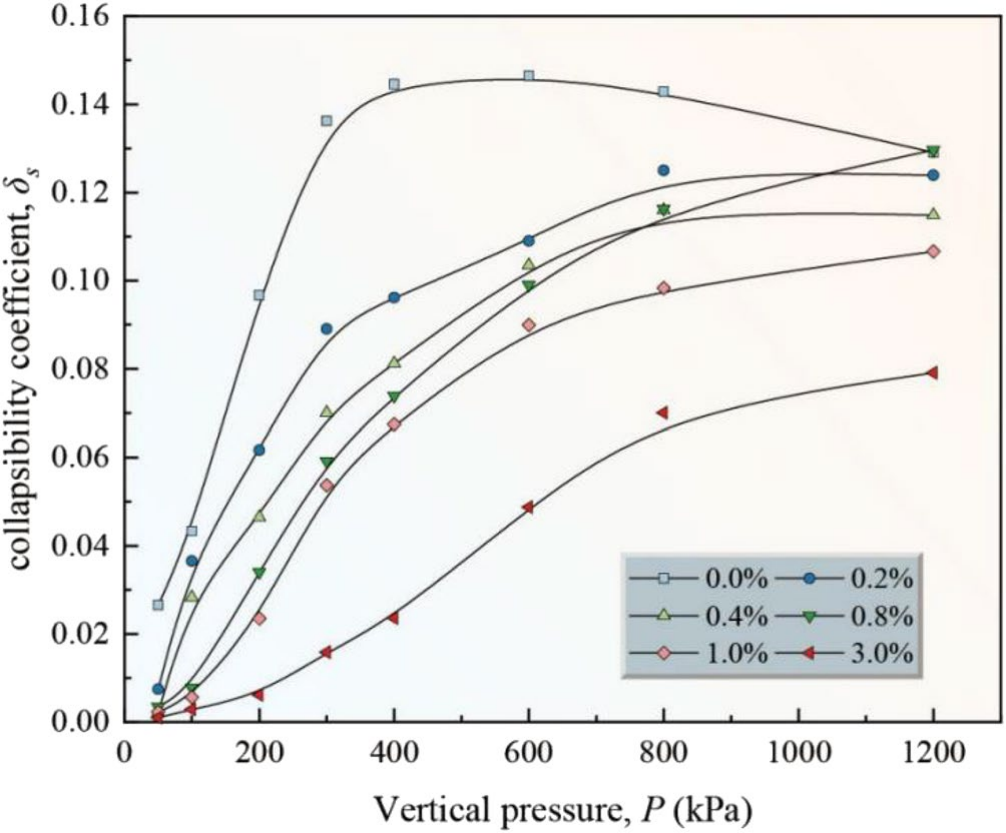
Relationship between collapsibility coefficient *δs* and vertical pressure *p* of loess samples with different nanosilica contents.

Figure 7 shows the relationship between the collapsibility coefficient *δs* and the vertical pressure *p* of the loess samples with different nanosilica contents. Two different trends are observed between the collapsibility coefficient *δs* and the vertical pressure *p* for the treated and untreated samples. (1) The collapsibility coefficient of the untreated loess increases and decreases with an increase in the vertical pressure. (2) The collapsibility coefficient of the treated loess samples increases and then stabilizes with an increase in the vertical pressure. The untreated sample has the highest collapsibility coefficient. The coefficient of the treated samples decreases with an increase in the nano-silica content. Other researchers have observed similar results^39,55^. However, they found an optimal nanosilica amount, whereas we did not observe this phenomenon^39^.

### Soil water characteristic curve

**Figure 8:**
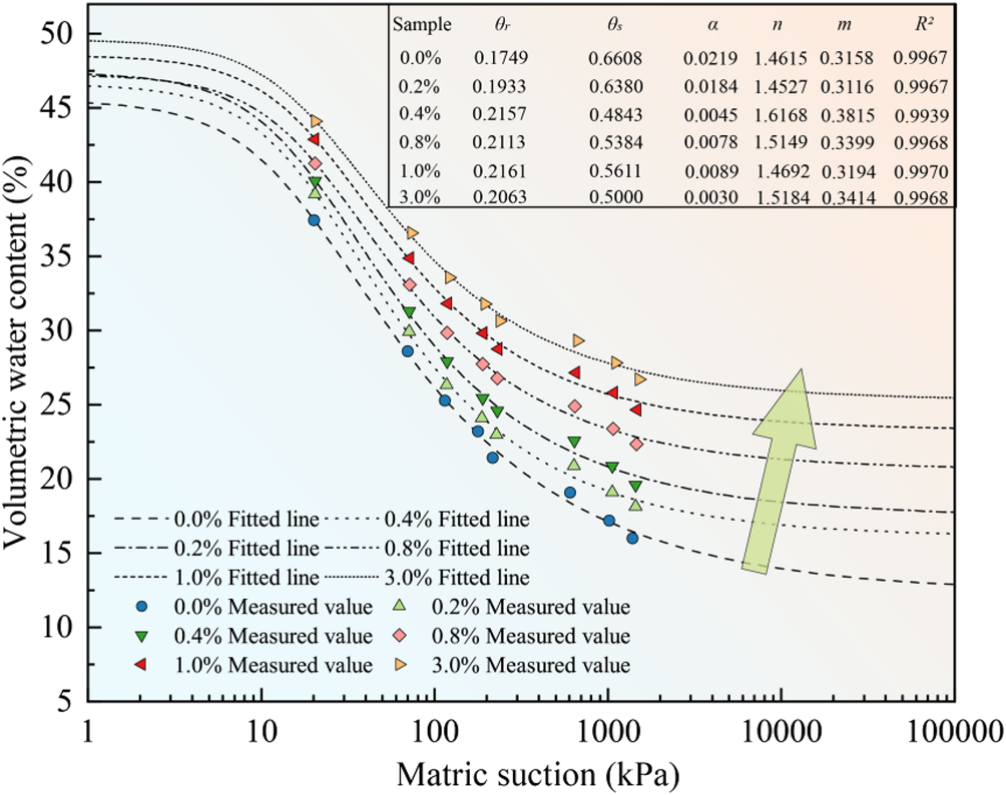
Soil water characteristic curves of loess samples with different nanosilica contents. Parameter values derived from the van Genuchten model.

Figure 8 shows the measured and fitted soil water characteristic curves of the loess samples with different nanosilica contents. The van Genuchten model was used to fit the curves. The fitting parameters and the fitting degree are listed in Fig. 8. A comparison of the fitted and measured curves and the fitting degree (*R*^2^ > 0.99) indicates that the van Genuchten model provides an accurate fit, demonstrating the result’s reliability.

The soil water characteristic curves of the loess samples with different nanosilica contents have similar shapes in the range of matric suction, and the volumetric water content shows a decreasing trend. At a matric suction of less than 20 kPa, the volumetric water content decreases slowly with an increase in the matric suction, indicating slow draining. When the matric suction exceeds 20 kPa, the volumetric water content of the loess decreases substantially with an increase in the matrix suction and then stabilizes. The loess sample with no nanosilica exhibits the fastest change, i.e., rapid drainage. The soil water characteristic curve shifts upward with the increasing nanosilica content, suggesting an increase in the water-holding capacity of the loess. This effect is more pronounced when the matric suction exceeds 200 kPa. The main reasons are as follows. (1) The soil water is primarily discharged from large and medium pores at higher water contents and from small and micro-pores at lower water contents. (2) An increase in the SSA of the particles enables more water absorption by the soil particles, increasing the soil’s water-holding capacity. (3) A decrease in the average particle size decreases the pore size and increases the force required for drainage^56^.

### Unsaturated permeability coefficient

**Figure 9:**
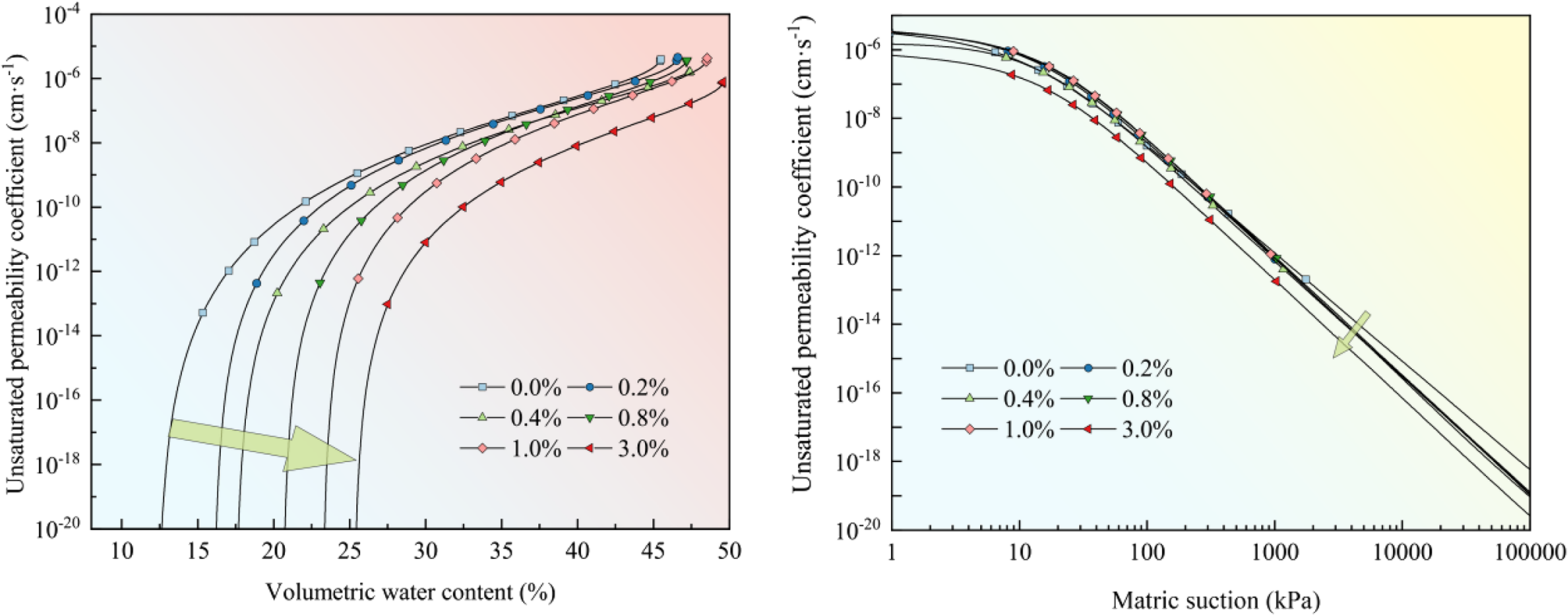
Unsaturated permeability coefficient of loess versus volumetric water content and matric suction.

Figure 9 shows the unsaturated permeability coefficient of the loess samples versus the volumetric water content and matric suction. The curves of the unsaturated permeability coefficient of the loess samples with different nanosilica contents have similar shapes. They show a nonlinear increasing trend with an increase in the volumetric water content and a nonlinear decreasing trend with an increase in matrix suction. The porosity of the soil decreases with the increasing nanosilica content. The larger pores are transformed into smaller pores as the nanosilica content increases; therefore, the permeability coefficient decreases.

### Microstructure

**Figure 10:**
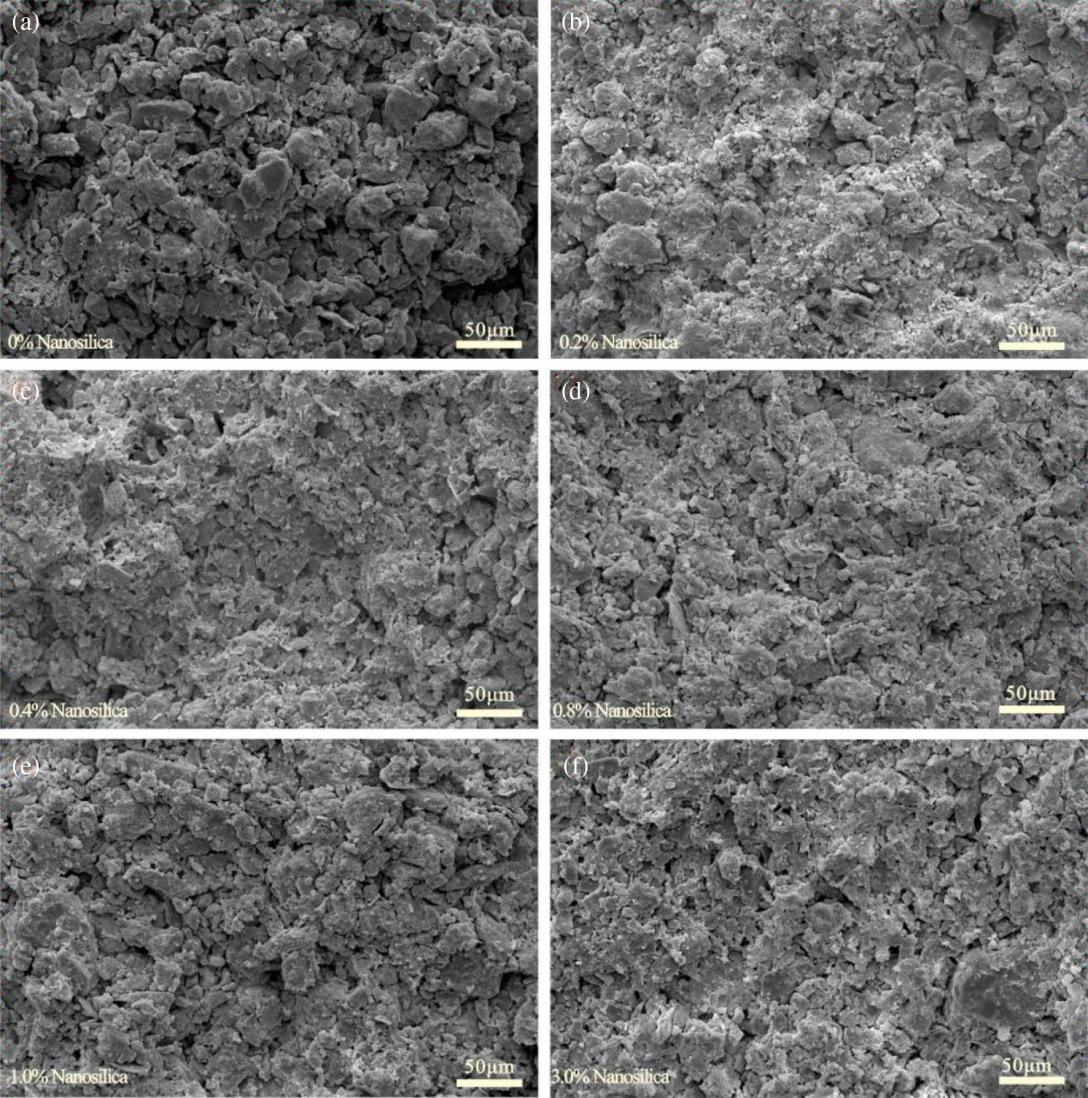
SEM image of loess samples with different nanosilica contents at 1000 times magnification.

**Figure 11:**
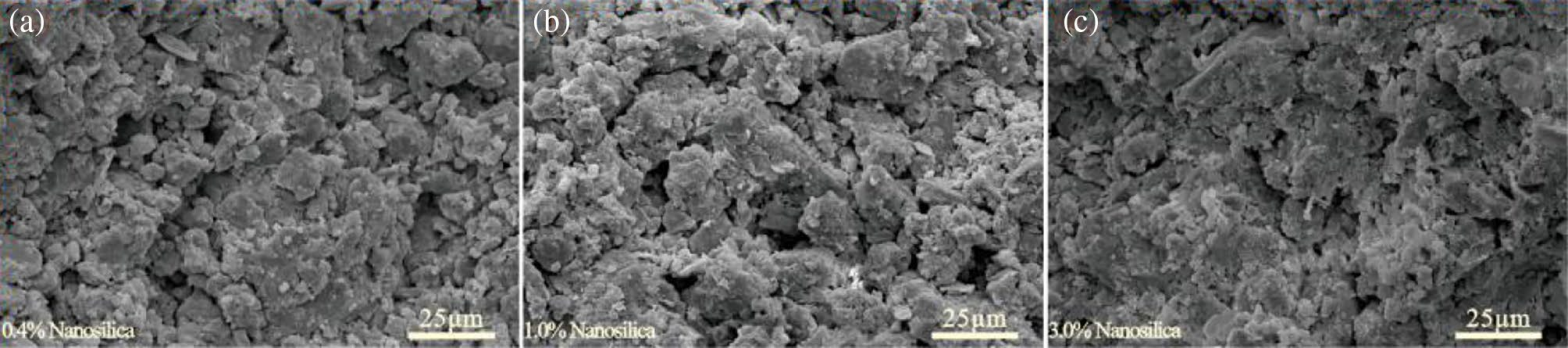
SEM image of loess samples with different nanosilica contents at 2000 times magnification.

The influence of soil physical properties (e.g., mineral composition and particle size distribution) on the macroscopic behavior of the soil manifests as changes in the microstructure^5,57–59^. Figure 10 shows the scanning electron microscope images of the loess samples with different nanosilica contents at 1000 times magnification. The edges of the loess particles are more defined, and large, medium, and small pores are clearly visible in the untreated samples. The porosity and pore connectivity are high (Fig. 10a). The pores between particles are partially filled, and the number of large and medium pores and the pore connectivity are lower in the samples with the nanosilica. As the nanosilica content increases, more fine particles are adsorbed onto the surface of coarse particles, and numerous micro-aggregates or clay clusters are visible in the pores between particles (Fig. 10b–f). Figure 11 shows that fine particles surround the loess particles, creating a coating that cements the particles together. The nanosilica particles aggregate with the loess particles and fill some of the large pores, decreasing the porosity of the loess and increasing the number of seepage channels. This finding is consistent with the results observed by Huang and Wang^60^ and Ghasabkolaei et al.^61^.

**Figure 12:**
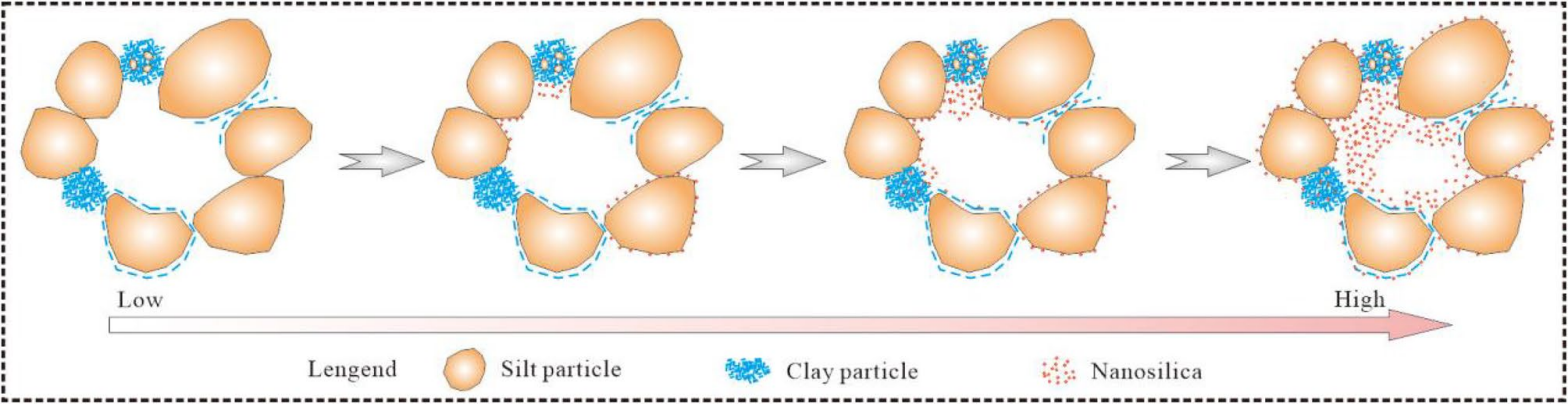
Conceptual model of loess microstructure after the addition of nanosilica.

The nanosilica does not react physically and chemically with the soil particles; thus, no new compounds are produced^38,42^. Therefore, the improvements in the hydrological properties of the loess by the nanosilica are attributed to its small size and large SSA. Even small amounts of nanosilica can have positive interactions with the loess particles due to the following mechanisms^23,33,36,60,61^. (1) After mixing loess and nanosilica with water, Ca^2+^ and Mg^2+^ ions will be produced, and Ca^2+^ and Mg^2+^ can replace K^+^ and Na^+^ ions adsorbed on the surface of clay particles, which makes the thickness of the double electric layer on the surface of clay particles thinner, that is, the binding water film forming a aggregate structure becomes thinner. Then the closer the clay particles are, the stronger the binding force between them will be; (2) Nanosilica is hydrophilic and has a large specific surface area, which can adsorb free water in the soil and make it become adsorbed water on the surface of the particles, thus reducing the content of free water in the soil; (3) The nanosilica becomes attached to the surface of the loess particles or covers silt particles or aggregate surfaces. It fills the pores and blocks the pore channels, reducing the porosity and pore connectivity of the loess; (4) The nanosilica and sub-micron colloidal clay particles in the loess contact and adsorb each other, forming small or micro clay aggregates. Their effect is similar to the clay components of the loess, i.e., the pores between larger aggregates are reduced, and the size of the small and micropores in the aggregate increases (Fig. 12).

## Conclusions

This study investigated the effect of nanosilica on the hydrological properties of loess and the microscopic mechanism. Analyses of the particle size distribution, Atterberg limits, soil water characteristics, collapsibility, and microstructure were conducted. The following conclusions were drawn:

1. The LL, PL, and PI of the loess increased after the addition of different amounts of nanosilica. The dominant particle sizes were 0.1–2 μm for clay and 2–50 μm for silt. The content of fine particles increased, and the content of coarse particles decreased slightly with the increasing nanosilica content in the range of 2–50 μm.
2. The collapsibility coefficient of the untreated loess increased and decreased, whereas that of the treated loess samples increased and stabilized as the vertical pressure increased. The collapsibility coefficient decreased with an increase in the nanosilica content.
3. The soil water characteristic curve shifted upward with an increase in the nanosilica content in the entire matric suction range, indicating an increase in the water-holding capacity of the loess. When the matric suction was less than 20 kPa, the volumetric water content decreased slowly with an increase in the matrix suction, indicating slow draining. When the matric suction exceeded 20 kPa, the volumetric water content of the loess decreased substantially with an increase in the matrix suction and stabilized. The untreated loess sample exhibited the fastest change, suggesting rapid drainage.
4. The unsaturated permeability coefficient curves of the loess samples with different nanosilica contents had similar shapes, showing a nonlinear increasing trend with increasing volumetric water content and a nonlinear decreasing trend with increasing matric suction.
5. The addition of nanosilica to the loess caused the filling of the pores between particles, a reduction in the number of large and medium pores, and a decrease in pore connectivity. As the nanosilica content increased, the nanosilica particles became attached to the surface of loess particles with various grain sizes and surrounded the silt particles or silt particle aggregates. The pores were filled, and the pore channels were blocked, reducing porosity and pore connectivity. The nanosilica and sub-micron colloidal clay particles contacted and adsorbed each other, forming small clay aggregates and micro-aggregates. Therefore, fewer pores existed between larger aggregates, and the size of the small and micro-pores increased. In addition, nanosilica can adsorb free water in the soil and make it become adsorbed water on the surface of the particles, thus reducing the content of free water in the soil.

## Data Availability

The datasets used and/or analyzed during the current study are available from the corresponding author on reasonable request.
